# Malignant upper urinary tract obstruction resulting in hospital admission: a qualitative study of patient, carer and clinician experiences and information received

**DOI:** 10.1136/bmjopen-2025-111467

**Published:** 2026-03-30

**Authors:** Tracey Stone, Jonathan Banks, Tim N Bond, Lucy E Selman, Chris Harding, Jonathan Aning

**Affiliations:** 1 National Institute for Health Research and Care, Applied Research Collaboration West, Bristol, UK; 2 University of Bristol, Population Health Sciences, Bristol Medical School, Bristol, UK; 3 Prospect, Bristol and District Prostate Cancer Support Group, Bristol, UK; 4 University of Bristol, Palliative and End of Life Care Research Group, Population Health Sciences, Bristol Medical School, Bristol, UK; 5 Department of Urology, Freeman Hospital, Newcastle upon Tyne Hospitals NHS Foundation Trust, Newcastle upon Tyne, UK; 6 Translational and Clinical Research Institute, Newcastle University Faculty of Medical Sciences, Newcastle upon Tyne, England, UK; 7 Bristol Urological Institute, North Bristol NHS Trust, Bristol, UK

**Keywords:** Adult oncology, Urological tumours, Patient Care Management, Interventional radiology, Urological tumours, Kidney & urinary tract disorders

## Abstract

**Abstract:**

**Objectives:**

Malignant upper urinary tract obstruction (MUUTO) is caused by advanced cancer. Developing MUUTO is often associated with approaching the end of life. Percutaneous nephrostomy (PCN) and retrograde ureteric stent insertion (RUS) are common interventions to treat patients with MUUTO, although neither intervention is likely to extend overall survival significantly. Little is known about patient, carer and healthcare professional (HCP) views of the MUUTO management pathway, the benefits and harms of the procedure and the treatment decision-making process. This study investigated the experiences, decision-making and priorities of patients admitted to hospital for MUUTO, and their carers, along with HCPs involved in providing care for this patient group.

**Design:**

Qualitative, using semi-structured interviews.

**Setting:**

This study was conducted across two NHS trusts in England.

**Participants:**

12 patients, 8 carers and 14 HCPs were interviewed. Patients were interviewed in hospital during their admission and, where possible, follow-up interviews took place 2–3 weeks later at their homes. In total, 18 patient interviews were conducted. Interviews were analysed thematically by cohort and systematically cross-referenced for areas of congruence and divergence of priorities and views. Ethical approval was obtained before study commencement.

**Results:**

Most patients were admitted as emergencies and received PCNs to relieve severe pain and distress. Patients reported having little choice in the decision-making around intervention due to their symptoms and frequently described the PCN procedure as being painful. HCPs considered the availability of further cancer treatment options a rationale to support intervening for MUUTO. However, HCPs reported decision-making was often complicated by unclear prognosis and the need to address the emergency nature of patient circumstances. A lack of compassionate communication, disrespect and indignity, traumatic hospital admission and premature discharge, in addition to practical administrative difficulties caused patient and carer distress.

**Conclusions:**

Emergency admissions for MUUTO are associated with significant patient and carer distress and are complex for HCPs to manage. MUUTO patients would benefit from a specific pathway to avoid emergency admissions and to facilitate timely advance care planning discussions so that patients’ wishes and HCP views can be shared and incorporated into decision-making about the appropriateness and value of PCN and RUS interventions.

**STRENGTHS AND LIMITATIONS OF THIS STUDY**

- This study explored the care pathway and decision-making for patients admitted to hospital with malignant upper urinary tract obstruction (MUUTO), an under-researched topic.
- Multiple stakeholders were recruited, including healthcare professional participants and meaningful insights were provided with direct implications for clinical practice around advance care planning, communication and pathway development.
- Interviewing patients and carers following discharge from hospital enabled a greater understanding of the experiences of people with MUUTO away from the emergency hospital environment.
- The number of patients receiving retrograde ureteric stent (RUS) in this study was limited; therefore, interpretations comparing percutaneous nephrostomy (PCN) and RUS should be made cautiously due to this imbalance.
- The results should be interpreted considering potential selection bias as the study only included individuals well enough to consent and be interviewed.

## Introduction

 Malignant upper urinary tract obstruction (MUUTO) may be caused by advanced cancer blocking the ureter of one or both kidneys, preventing urine flow and causing kidney failure. Treatment of MUUTO aims to enable kidney drainage, preserve or improve renal function and improve quality of life and overall survival.^1^

Percutaneous nephrostomy (PCN), where a tube is placed through the patient’s skin into the kidney and retrograde ureteric stent insertion (RUS), where a tube is placed internally through the natural passageways from the bladder into the kidney, are the most common minimally invasive interventions used to treat MUUTO.^1^ There is a lack of consensus among clinicians regarding the optimal method of dis-obstruction. Both PCN and RUS have a significant associated risk of complications, readmission and need for ongoing management burden which negatively impacts on quality of life.^2^,^5^ The benefits and harms of treating MUUTO can be uncertain, particularly in patients approaching the end of life, with the current evidence base limited by a high prevalence of studies of poor methodological quality.^1^

Few qualitative studies have investigated patients’ experiences and perceptions of their MUUTO management pathway and none have examined healthcare practitioners’ perspectives of MUUTO management.^6^,^8^ There is a need to better understand the patients, carers and clinician priorities and needs of those involved in the MUUTO decision-making process and management pathway, to inform clinical practice and policy. This study aimed to address this evidential gap by evaluating the experience of patients admitted to hospital with MUUTO, carers and healthcare professionals (HCPs) drawn from two geographically diverse UK hospitals.

## Methods

### Design, setting and inclusion criteria

This prospective qualitative study employed face-to-face semi-structured interviews. The research was based at two hospital trusts in the West of England (site 1) and the North East of England (site 2). Patients over 18 years, with the capacity to consent, admitted with a first diagnosis of previously untreated MUUTO to either NHS trust during the study period (April 2023 to November 2023) were eligible for inclusion. Carers for patients with MUUTO and HCPs involved in the MUUTO management pathway at each NHS trust were also eligible for inclusion. Recruitment of participants took place sequentially at site 1 followed by site 2. Patients without capacity to consent were excluded from this study.

### Patient and public involvement

A member of the prostate cancer support group, local to site 1, is a member of the project management group and has been involved from pre-funding to dissemination. He and four other patient and public involvement (PPI) members provided input into the study design, topic guides and the design of patient and clinician facing materials at two PPI meetings facilitated by TS.

### Sampling and recruitment

The recruitment strategy for patients was driven by convenience sampling and included male and female patients. Potentially eligible patients were assessed by research nurses at each site to determine whether they were well enough and had capacity to consent and participate. The research nurses provided eligible patients with the study patient information sheet. The research nurses then contacted TS to arrange a convenient time for interviews with patients who agreed to participate. Interviews took place between 12 hours and 2 days following the PCN or RUS procedures or within a similar time frame from the point at which patients had decided to decline the procedure. Patients were invited to do a follow-up interview 2–3 weeks following the procedure to capture their reflections on the impact of the PCN or RUS on their quality of life. Patients were recruited until the research team were satisfied that the concept of ‘information power’^9^ had been achieved. Carers of recruited patients were also invited to participate in an interview.

Clinicians were sampled using maximum diversity sampling to ensure that roles were recruited from across the spectrum of HCPs involved in the MUUTO treatment decision-making process, the surgery and the ongoing treatment associated with PCN and RUS (specialist nurses, oncologists, urologists, interventional radiologists, radiographers and palliative care consultants).

### Data collection

Topic guides (see online supplemental files) for the three groups (patients, carers and clinicians) were developed by the research team, based on the literature and clinical experience and reviewed by the study’s PPI group.

Initial patient interviews took place in hospital. Follow-up patient interviews were performed in patients’ homes, or by telephone, 2–3 weeks later. Carer interviews were by telephone or online, as preferred by the participant. Clinician interviews were online or by phone. Written informed consent was obtained prior to data collection.

### Data analysis

Interviews were audio-recorded, transcribed and anonymised before importing into NVivo QSR V.12. Analysis was developed by TS and supported by JB, both experienced qualitative researchers. Coding frames for patients, carers and staff were developed from early readings of the transcripts and one early interview from each cohort was independently coded by JB and TS. Data were analysed thematically by TS and JB using an inductive approach.^10^ The same six-stage process was applied to the raw data for each of the three cohorts. Stage 1: Initial ‘immersion’ by close and repeated reading of the transcripts was followed by Stage 2: the generation of initial codes, including deductive codes in line with the research question and topic guide. As inductive insights arose, they were coded where they added insight to deductive codes, or as new codes where they provided alternative perspectives. Stage 3: Codes were then collated into themes by virtue of their relative importance to participants and to the research question and, Stage 4, reviewed by JB and TS. Stage 5: The coding frames were subsequently developed and agreed after which TS coded the full dataset in NVivo QSR V.12 with regular analysis discussions with JB to refine and define each theme. For example, the theme of ‘pain’ was pertinent, in differing ways, to all three cohorts though subcodes pertaining to what was important for each held categories of meaning that differed but sometimes overlapped. The data were initially analysed by interviewee group before systematically cross-referencing to identify areas of agreement and divergence. Results were reviewed by and discussed with the research team (clinical Principal Investigator (PI), subject specialist researcher, patient representative and researchers) during the analysis. Stage 6: The manuscript was prepared following the COREQ (Consolidated criteria for Reporting Qualitative research) reporting guidelines.

### Participant quotation coding scheme

Illustrative quotations below anonymously identify participants as follows: Pat=patient, Carer=carer and Clin=Clinician. The participant descriptor is followed by the participant number, and the location where the interview took place, either in hospital=hosp or at home=home. For clinicians the specialty of the clinician follows their participant number, the participants specialty is summarised as follows: Urology=urol, Oncology=oncol, Palliative care=pal, Clinical Specialist Nurse=*ClinSpNrs*. For example, (*Pat4-home*) describes: patient number 4 interviewed at home.

## Results

### Study participants

In total, there were 34 participants: 12 patients, 8 carers and 14 HCPs. Of these, 10 patients and 7 HCPs were recruited from site 1 and 2 patients and 7 HCPs from site 2. One patient participant was black, the remainder were white. Follow-up interviews at home were performed with 6 out of the 10 patients who underwent their first interview in hospital; the remainder were unresponsive to contact after discharge. Two patients were discharged before an interview was possible and were both interviewed once, at home. Table 1 presents patient and carer participants’ demographic and clinical characteristics. Of the 12 patient participants interviewed, 10 underwent PCN, 1 underwent RUS and 1 declined intervention. Table 2 describes the clinician participants’ characteristics.

**Table 1:** Patient and carer characteristics

Patient	Gender and age (approx.)	Admission	Primary disease	Procedure	Treatment post intervention	Interview 1	Interview 2	Carer interview
1	Male 60s	Emergency	Prostate cancer	PCN	Yes	Yes	Yes	No
2	Female 50s	Emergency	Bladder cancer	PCN	Yes	No	Yes	White male 50s
3	Male 80s	Emergency	Bladder cancer	PCN	No	Yes	Yes	White female 40s
4	Male 80s	Emergency	Kidney cancer	None	No	No	Yes	White female 70s
5	Male 80s	Emergency	Kidney cancer*	PCN	Yes	Yes	Yes	White male 50s
6	Male 70s	Emergency	Kidney cancer*	PCN	Yes	Yes	No	No
7	Female 80s	Emergency	Breast cancer	PCN (GA)	No	Yes	No	White male 50s
8	Female 80s	Emergency	Bowel cancer	PCN	No	Yes	Yes	White female 50s
9	Male 80s	Emergency	Prostate cancer*	PCN	Yes	Yes	Yes	White female 50s
10	Female 50s	Emergency	Rectal cancer	PCN	No	Yes	No	No
11	Male 80s	Elective	Bowel cancer	RUS (GA)	Yes	Yes	Yes	White female 70s
12	Male 50s	Elective	Rectal cancer*	PCN	Yes	Yes	No	No

* Malignant upper tract obstruction diagnosed at new presentation with cancer.

GA, general anaesthetic; PCN, percutaneous nephrostomy; RUS, retrograde ureteric stent.

**Table 2:** Clinician characteristics

Clinician	Specialty	Role
1	Palliative care	Consultant
2	Urology	Consultant
3	Interventional radiology	Consultant
4	Oncology (urology)	Consultant
5	Radiography	Senior radiographer
6	Urology	Senior nurse specialist
7	Oncology (gynaecology)	Consultant
8	Interventional radiology	Consultant
9	Oncology (urology)	Consultant
10	Oncology (urology)	Consultant
11	Urology nurse specialist	Senior nurse
12	Palliative care	Consultant
13	Palliative care	Consultant
14	Urology	Consultant

### Findings

The results are presented around the pathway to PCN/RUS in the following themes: Prognostic uncertainty, PCN and RUS—the rationale, Relieving acute distress, Alternative decisions to PCN/RUS, Decision-making and patient involvement, Decision-making and clinical support, Experience of the PCN/RUS procedure, Living with PCN/RUS, The role of palliative care and advance care planning.

#### Prognostic uncertainty

Patients who are considered for PCN/RUS enter the pathway with a high degree of prognostic uncertainty. Many patients reported that rapid disease progression often coincided with their MUUTO, especially when they were admitted as an emergency. This was confirmed by clinician accounts which also described that there was little time in the emergency situation to establish what the patient knew about their life expectancy or to update them on any negative developments. Patients highlighted that various clinical teams would be involved in their care any of whom may or may not have had a conversation about prognosis. While clinicians sought an understanding of the patients condition between themselves, if, when and by whom this had been communicated by the clinical team to the patient was not always known, did not happen or was done ineffectively according to both clinicians and patients:

(I)t’s interesting, you’re discussing with oncologists and other people that sometimes patients don’t realise quite how bad things are…they’ve been under a gynaecologist or oncologist having lots of treatment but no-one’s ever actually said to them you’ve not really got very much longer and then suddenly you’re in a situation where you’ve gotta try and speak to them and explain that… And whether they have done and the patient’s just not taken that on board… you can’t tell. But that’s not infrequent that we then end up being the bearer of bad tidings. (Clin14-urol)

Patients were deeply concerned and confused about the deterioration in their health that led to MUUTO and the implications for their life expectancy. Usually scans and other investigations performed to identify the obstructed kidney would reveal the degree of malignancy or metastases, but patients reported being left confused about the implications of these. They reported rarely seeing the same doctor and not knowing the doctor’s role in relation to their treatment. They often struggled to make sense of different messages from different teams. Patients were particularly panicked by medical uncertainty and how it was communicated to them. Almost all patients were upset by being unable to develop a clear story about what was happening to them.

I was in ((Hospital1)) about a day, day and a half and one of the doctors and about five other doctors came into my room and they gave me the devastating news that I’d got a fortnight to six months to live! …They didn’t look much like nurses to me. They were all in there - well like the other doctors were dressed I suppose. Like the one that was talking to me…He just came out with it just so boldly and bluntly in front of all of us. And then he said a few more words, and they all trailed off and left me sat there on the bed on my own. (Pat4-home)

Clinicians acknowledged the difficulty of patient-centred communication when multiple teams were working with unpredictable cancer progression in a pressured environment:

You’re always on the back foot and it’s difficult. Sometimes, for both doctor and patient to keep up… and things can change very quickly and that’s very difficult for both patient and their medical teams. (Clin10-oncol)

Clinicians were also aware of the possibility of patients receiving ‘mixed messages’ from different professionals, and how harmful this can be.

I think the worst thing that you could do would be to mix the message for the patient, whereby one professional is saying one thing and other people are saying different things, which sometimes happens too. (Clin12-pal)

#### PCN and RUS—the rationale

The main reasons outlined by clinicians for offering PCN/RUS were: to enable further treatment, to relieve extreme pain and suffering or to avoid life-threatening sepsis, with the latter two aspects particularly associated with emergency admissions. Clinicians explained that undertaking PCN/RUS could also create the time and space needed to consider further treatment decisions or they could enable a patient to leave hospital and die at home if they wish:

When you come into the end of life, no other option, you just look at them, are they dying? Are they symptomatic? Do I need to do something to help with the situation? Is the procedure going to be too much a risk in resulting of dying early? Will the patient survive this anaesthetic (RUS) or the procedure. Those sort of questions are quite basic. (Clin9-oncol)

Having further treatment options, including chemotherapy, palliation drugs and clinical trial options, was reported as a key driver in clinicians encouraging patients towards PCN/RUS. However, it was noted that future treatment did not always materialise:

It’s slightly frustrating from our point of view when you perhaps have that conversation with the oncologist who says ‘oh, yes, we’re gonna give treatment’ and then when we’ve looked back and audited it, even though they say that, actually a lot of patients don’t ever get further treatment. (Clin14-urol)

Where further treatment options were not available to patients with MUUTO clinicians described not recommending intervention with PCN or RUS:

Unless we’ve got a definite aim for further treatment or improvement in quality of life or length of life, then we would generally counsel them that we wouldn’t want this to happen. (Clin4-oncol)

#### Relieving acute distress

Clinicians reported that when patients arrived acutely unwell, clinical decision-making focused on making a ‘best interests’ decision for the patient, particularly when the patient lacks the capacity to make a decision. At site 1, patient participants were mostly emergency admissions and reported extreme pain. Clinicians reported this as a rationale to perform PCN or RUS:

If they really are that sore, if the only way of managing the pain is drainage then you have to drain and to say then it doesn’t matter whether they’ve got a week to live or six months. If you’re not managing with decent analgesia then drainage is what you have to do. (Clin14-urol)

In these situations, clinicians feel that decision-making is removed from them and they are ‘forced’ to intervene:

These are patients that we are absolutely not trying to intervene unless we are, if you like, forced to and particularly when the intervention is not one that is… it’s one that’s not comfortable, carries risk and has long term consequences, in terms of requiring nephrostomies and things. (Clin10-oncol)

This sense of having no alternative was also expressed by patients:

Well, in a way I don’t suppose I had a choice. I wasn’t offered a – I suppose I could have said, no, I don’t want that but what would have happened then? I don’t know. (Pat5-hosp)

It was acknowledged that emergency admissions for decompression of the kidney were stressful for clinicians and patients alike:

I think sometimes the difficulty can be that, even if you feel it’s the wrong thing to do, because you’re making an emergency or sort of relatively quick decision-making, the patient doesn’t always have time to reflect on that and have those big discussions … on the spot, patients don’t want to die. (Clin2-urol)

I virtually came in here and went straight to the procedure, where they did this nephoscopy, which I had no idea really, they explained but …anyway, I had it done and was brought back to here and just slept all night… Well, they told me they’d put a stent in to relieve the pressure. I didn’t know much about it really, not at all. And quite frankly I didn’t care, I think by the time you’re in that situation you just want relief. (Pat8-hosp)

#### Alternative decisions to PCN/RUS

Clinicians and patients have to consider the negative aspects of intervention in their decision-making. In these circumstances, patients being considered for this procedure are often nearing the end of life. All of those involved in the decision-making take into consideration quality of life issues in the time remaining for a patient and balance those against the potential benefits, drawbacks, likely outcomes and side effects of the intervention. For a patient who may already be suffering from the symptoms of their cancer the intervention represents a significant short-term burden, with uncertain benefits.

(I)t’s not nice to think of these patients who are sometimes already in a lot of pain because of disease progression or whatever, then having to endure a 30 or 40 minute procedure that is pretty gruelling. You know, I’ve seen a nephrostomy inserted and it’s - you wouldn’t like to wish it on yourself or a loved one. (Clin11-ClinSpNrsurol)

Deciding not to intervene involves thinking about the impact of the intervention versus what constitutes a good death for that patient:

To slip away with your kidneys failing is a very kind and gentle death. You literally just slip away. You can become toxic, which isn’t necessarily nice for your family so you don’t become angry or anything but you might become a bit confused. (Clin11-ClinSpNrsurol)

I’m very happy with the decision I made because I don’t want any more. I just want a quality of life, you know. I don’t want to…. I have spoken to my GP about it, yes and the kidney doctor we saw yesterday and she agrees with me that it was the right decision. (Pat4-home)

#### Decision-making and patient involvement

Where patients had capacity, clinicians reported that they aimed to ensure that the patient was the decision-maker.

It’s always the patient’s choice to accept the treatment, it’s not mine… The patient is very involved… typically if you say ‘your kidneys aren’t working we need to try and fix them so we can give you chemo more safely’ they don’t say no. (Clin7-oncol)

Clinicians reported that relatives were involved in discussions where this was possible, more usually with RUS, in non-emergency situations, where more time was available and the patient wanted this. Occasional cases of disagreement between patient and family were said to be navigated by the clinician to enable the wishes of the patient.

(I)t’s often daughters and dads… the dad might say, that’s not what I want, and the daughter is saying, no you have to… that’s just their instinct to keep their dad alive and I think it’s normally quite easy. Peace is the wrong word, but I think you can resolve that through conversation and it’s all part of the acceptance process for the family. (Clin9-oncol)

Involving carers for emergency admissions was not always possible as they may have been travelling or taking a break when treatment was proposed.

I wasn’t there when she had the conversation about the nephrostomy. She told me about that and no-one spoke to me separately about it or explained it…we thought it was going to be major surgery and of course it’s not. (Carer8)

Most patients interviewed felt the decision was theirs.

Oh yes, I did have a choice, yes. So there was no doubt about that. (Pat5-hosp)

No written information was offered in support of decision-making, other than the consent form, although at site 1 patient information leaflets describing the procedure were available on the ward. Rather, an understanding of what was being proposed was relayed verbally by the clinician and under the emergency circumstances patients were content with this.

(T)hey were very thorough in explaining what would happen. I had it twice. Effectively the two people, the two doctors, the one I already knew, he came in and talked to me about it. Said, ‘Do you want to know about everything?’ I said, ‘Yes, I would really, please.’ He said, ‘This is what we plan to do.’ Then when I came in, the other one went through the same thing in great detail, so I’m very pleased with that ‘cause I like that. I don’t go’, ‘I don’t want to know, just do it.’ I’d rather know what are you – even if it’s horrible, I’d rather know, so I know what to expect. (Pat3-hosp)

However, patient involvement in decision-making could be perceived as problematic for emergency admissions where time was short and decisions had to be made quickly:

It’s a time sensitive thing, but yet we’re recognizing the importance of patients having as much and full information and time and space to digest it as possible. So those tensions exist. (Clin13-pal)

As noted above, for most patients admitted as emergencies they felt that they had no choice but to consent because they were so unwell.

I didn’t care what they did at the time because I was in so much pain. (Pat7-hosp)

The one patient who declined a nephrostomy was also an emergency admission but felt in a position to weigh up options and make an informed decision, and in this case, they were able to get symptom relief shortly afterwards with successful catheter placement.

(T)hey said we can put a, I forget what the word they is to say, but we can put it into your kidney to sort of help keep it unblocked and it meant having an operation. And I thought that the amount of things that I’d had done and the way that I felt and the way that the doctor spoke to me, I didn’t want any more invasions, that’s the word I was looking for. So, you know, I decided against it. …I just told them that I didn’t want it and I didn’t find it difficult to tell them. (Pat4-home)

#### Decision-making and clinical support

Clinicians identified good and accessible collegiate relationships as a key resource in decision-making with access and proximity to colleagues making this easier. Decisions about whether the patient is offered PCN/RUS at all tended to be made between the clinician with primary responsibility for the patient and senior colleagues in the specialties involved.

(A) lot of these conversations will happen in the corridor or in the – there’s a room where everybody sits and does all the documentation. The oncologists like, “Oh, (Clin13), can I talk this through?” Or, “I’ve got this patient, can you come see them with me?”, so it’s just very natural,… I’m very lucky to have that relationship. (Clin13-pal)

Participating clinicians felt that decision-making for PCN/RUS procedures were too nuanced and time sensitive to be discussed at multidisciplinary-team (MDT) meetings. Rather, informal discussions with other clinicians treating the patient were more helpful.

I would never take this sort of situation to an MDT. (Clin10-oncol).

Despite having support from colleagues, clinicians still felt a degree of uncertainty about whether PCN/RUS was appropriate.

(M)y preferred style is for that to be a shared decision between myself with the palliative care experience, an oncologist, if the person has one, and then the urology team who would be inserting a nephrostomy …I was often left with the impression that something could be done, but we didn’t have the answer to the question of should it be done. (Clin12-pal)

In some cases, patients who were end of life, with no further treatment options, not in acute renal distress and pain and deemed too frail to travel or undergo the procedure were effectively ‘screened out’. This is a difficult ‘best interests’ decision to make and especially so in emergency situations.

(S)ometimes they wouldn’t even come to an oncologist, to be honest, because the frail or elderly, they’re may be in a ward and they may be seen by palliative care and the decisions made for symptomatic care. (Clin4-oncol)

There are currently no clinical guidelines specifically for decision-making for PCN/RUS procedures with patients towards the end of life with advanced disease. Discussion with colleagues close to the patient, and the patients themselves, was reported as, potentially, more useful than what guidelines could offer.

Personally, I think we’ve got too many guidelines, and this sort of thing can be quite rigid … I think everything is holistic, everything is a combination of factors and joint decision-making, clinical and personal, and disease status. (Clin9-oncol)

However, some clinicians favoured the development of decision support tools which would account for frailty, comorbidities and disease stage to give prognostic information.

The most helpful thing would be clarity on working, … a national database… so we had some clearer data on the prognostic indicators. which patients are going to do better…blood test, fitness, frailty scores, BMIs, whatever, it might be you put all that into an algorithm. (Clin14-urol)

#### Experience of the PCN/RUS procedure

Clinicians reported that patients varied in their capacity to withstand the discomfort of the procedures and that this was difficult to predict. Initial nephrostomy usually caused the greatest distress, stent placement caused unpleasant pressure during the procedure or as a secondary procedure to initial nephrostomy. RUS is done under general anaesthetic (GA) so the patient reported no memory of the event.

Clinicians noted that PCN and stenting is often painful but that GA is not necessary, or possible in patients who are likely to have other compromised organs.

It’s not that nice a procedure so patients generally don’t like it very much. Although they do give them pain relief and local anaesthetic, a lot of them say they wouldn’t be keen to have another one. I think sometimes we do underestimate a little bit the actual procedure itself. (Clin2-urol)

Patient experience was mixed, with some finding the procedure reasonably comfortable.

A little bit painful but it was okay … it was reasonably painless, I could tolerate that again, that’s no problem. (Pat9-hosp)

However, the patients who did experience difficulty described extreme pain. Many patients were surprised by how painful the procedure was and had not understood this at the time of giving informed consent.

They put temporary tubes in my back, one into each kidney, this side was really, really painful. I thought, ‘Oh, God, I hope I’m not going to have something like that again. (Pat3-hosp)

#### Living with PCN/RUS

Immediately post-procedure, patients described symptomatic relief and feeling much better. Despite being grateful for the resolution of severe pain, they were all weak, tired and confused by their condition and what was happening next, with varying degrees of physical discomfort at interview. They also had trouble adapting to the nephrostomy bags, with reports of leaking and worries about getting them changed and how to deal with the bags themselves.

The real problem is, who was going to change this (bag) and when, because the last two nights I’ve woken up drenched… …So that’s happened two or three times running… I’ve never experienced that before and there hasn’t been, funnily enough, a regular, you know, how’s your bag?…which would have been good. (Pat5-hosp)

A specialist hospital-based nurse highlighted the challenges of living with a nephrostomy.

They stick out your back at a funny angle (PCN drain) and you can’t lay comfortably, and you can’t roll over, they’re very rigid in the back sticking right out…(S)tents are horrible. Stents are just the devil’s spawn. It’s horrible even caring for people that are dying with stents in because even when they’re semi-conscious you can still tell they’re in pain because they hold themselves in their genitals because they’re so uncomfortable and it’s horrible. (Clin6-SpNrsurol)

Discharge from hospital was often confusing, with patients and carers uncertain of ongoing care and treatment provision, bags and supplies, their prognosis and the implications of having had a blocked kidney for the progression of their disease.

I do realise that if it wasn’t for my daughter I would be totally up the creak…mainly because the information that came with me home was just not adequate at all…if ((daughter)) hadn’t been with me I think I’d have come home without any bags to change …It was very hit and miss. (Pat9-home)

Specialist nurses felt that support in the community was lacking.

District nurses aren’t joyful about nephrostomies… they’re scared of them. “It’s unfamiliar, it’s something that they’re a bit freaked out by”. (Clin6-SpNrsurol)

#### The role of palliative care and advance care planning

The palliative care doctors interviewed acknowledged that having a conversation with patients about having no active curative treatment options remaining is difficult, and may often be avoided or done badly by clinicians. They were keen to support both patients and colleagues by being involved in those conversations where possible and by offering learning opportunities for colleagues about how to manage such conversations well.

it’s important for urologists to take up [conversations]. If they are the ones who are mostly going to be having these decisions and these discussions before they involve us, is for them to just really focus on the developing their skills, their nuance skills of recognising where these people are in their disease and recognising that they need to be able to have those discussions … [and] to develop more joint working, with more education for them on these particular issues or even speaking to us and just brainstorming. (Clin1-pal)

The idea of discussing patients’ treatment preferences around PCN and RUS much earlier was mooted by clinicians. Kidney obstruction is very common in some cancers and this was felt to be a good reason to include a consideration of the possibility of obstruction and how it might be managed well before patients presented in emergency situations.

They would often ask what symptoms they might get, so then you can go onto different assistance depending on what disease states, and kidney failure is a very common pathway. (Clin9-oncol)

## Discussion

### Summary

This study presents the first, to our knowledge, in-depth qualitative investigation of the journey to MUUTO treatment through the experiences of patients, carers and HCPs. For our patient group, there was no defined pathway and consequently patients frequently described a difficult path to hospital admission with MUUTO, often felt vulnerable and experienced a significant amount of uncertainty regarding their prognosis. Patients experienced a lack of consistency in management approaches and reported being reviewed by a range of clinicians. Patients and carers described a general lack of information available about MUUTO aftercare and a need for this to be urgently addressed. On discharge, many patients felt unsupported and unclear about their future. Their carers reiterated this view, stating that often they were not present at or involved in MUUTO treatment decision-making conversations and then after discharge were left ‘picking up the pieces’ of support in the community with little information. HCPs perceived a clear rationale for intervening to treat MUUTO if there was clear evidence that patients would receive further oncological treatment or symptomatic improvement in terms of relief of pain or infection could be achieved. However, HCPs felt conflicted in the absence of a clear indication to intervene because of uncertainty about prognosis. Frustration and worry were experienced by admitting clinicians unfamiliar with patients, when patients were admitted as an emergency, regarding the risks of advising or supporting patients to make the ‘wrong’ decision. Patients, HCPs and carers all concurred that emergency presentations with MUUTO should be avoided if possible as this led to complex presentations with patients potentially having impaired capacity to make a pressured decision.

### Strengths and limitations

Our study limitations include that the study was conducted in a specific cultural and medical context in two centres in the UK and the findings may not be transferrable to other locations. We acknowledge that our study findings may be subject to selection bias because having sufficient competency to provide informed consent and being physically able to participate without distress were ethical prerequisites for patient participants. This means that we were unable to include the views of patients who were the most unwell, or their carers, who may have contributed differing perspectives. Though clinicians may have indirectly represented the views of these patients, the results should be interpreted in light of this limitation. The number of patients interviewed who had undergone RUS was low. More RUS procedures were undertaken in site 2; however, due to geographical distance and swift hospital discharge for the patients, the researcher was unable to respond to potential interviewer-interviewee invites in a timely fashion. We believe, however, that the strengths of our study outweigh these limitations, as findings provide, for the first time as far as we are aware, an in-depth understanding of key perspectives and experiences from those involved in the procedures to alleviate MUUTO. We did not return interview transcripts to participants for participant validation. There was consensus within our PPI group which informed the study design, that we should not burden MUUTO patients or their carers inappropriately when they were significantly unwell and approaching the end of life. It was felt that any potential gain in validity would not compensate or justify any distress caused by extended communications. Additionally, recall bias in our sample was minimised in comparison to previous qualitative studies in this area because patients were interviewed in hospital around their treatment and most patients were also interviewed at home a short while later, which enabled reflection on the value of the intervention in terms of their quality of life.

### Comparison with existing literature

Similar to other studies in acute surgery, patients felt that they had little choice under the emergency circumstances, given their distress and discomfort, and were content with verbal explanations for the procedure itself.^11 12^ Other qualitative studies performed to date have focused on the impact of MUUTO and specifically intervention to relieve obstruction from a quality of life perspective after intervention, and generally included patients who had a relatively long survival. Bigum *et al* investigated how PCN is perceived by male patients with urological cancer and the implications on everyday life.^6^ They found that most patients did not fully understand the later impact of their MUUTO treatment until it became a reality. Similar to our findings, Bigum *et al* found that there was a lack of follow-up after PCN and a lack of information about how to look after the tubes and dressings for the patient, carers and home care nurses. Longer-term, the burden of living with PCN was determined and the authors found that if patients did not perceive that their life was saved by the intervention then the burden of misery and despair caused by the PCN was more.^6^ Kumar *et al* investigated the impact of ureteral obstruction relief on quality of life in patients with gynaecological, urologic and colorectal malignancies. In line with our findings, and that of Bigum *et al*, an educational void was demonstrated with regards to looking after stented or urinary diversion patients and this was described as aggravating post-procedure symptoms. Difficult symptoms were experienced by patients after stenting or diversion procedures.^7^ Our study has further illuminated these findings adding an understanding of the perspectives of carers and HCPs. Pure quality of life studies in patients undergoing palliative procedures for malignancy-related urinary tract obstruction using validated questionnaires such as Functional Assessment of Cancer Therapy–General and Bladder over a 16-month period suggest that quality of life is not significantly different between PCNs and ureteric stents at 7, 30 and 90 days regardless of procedure.^8^ Patients undergoing double jj stents had a greater incidence of urinary symptoms and pain and those with nephrostomy had more frequent tube changes after intervention.^8^ In our study, the number of patients undergoing RUS was limited, so interpretations comparing PCN and RUS experiences should be made cautiously. Nevertheless, the data from our study contextualises the real-world experiences of patients with MUUTO and highlights the specific limitations of the present pathway that cannot be picked up in these questionnaires but need to urgently be addressed.

### Implications for research and practice

There is a need for research in defined primary malignancies investigating the benefit and optimal timing of MUUTO decompression. Data from well-designed studies incorporating patient assessment of the burden of intervention is required to better inform clinician counselling and practice. In the interim, though, our findings demonstrate a clear need for better advance care planning, information provision and pathways for patients with MUUTO. The development of prognostic tools for MUUTO will support advance planning,^13^ but there is also a need for a consistent management pathway for patients with MUUTO underpinned by the following standards in care for patients with MUUTO: (1) pre-empting MUUTO should be part of the discussion at advanced cancer diagnosis discussions; (2) providing patients with clear, consistent and accurate information about their treatment options is a key requirement for adequate shared decision-making. There is an urgent need for resources to be developed. Ideally, this information should have been shared prior to or at emergency presentation; (3) discussing the end of life and treatment options is a skill that all clinicians should have and not solely specialist palliative care physicians. Clinicians may benefit from communication training with palliative care colleagues as this acute event often indicates disease progression and reduced life expectancy.

## Conclusions

Patients’, carers’ and HCPs’ experiences demonstrate that there is an urgent need to improve the pathway for patients with MUUTO. To improve the shared decision-making process, advance care planning and compassionate, accurate information provision is crucial. Upskilling all clinicians to be comfortable discussing end-of-life care is essential to ensure that all patients receive a consistent, patient-centred approach.

## Supplementary material

10.1136/bmjopen-2025-111467 online supplemental file 1

10.1136/bmjopen-2025-111467 online supplemental file 2

10.1136/bmjopen-2025-111467 online supplemental file 3

10.1136/bmjopen-2025-111467 online supplemental file 4

10.1136/bmjopen-2025-111467 online supplemental file 5

10.1136/bmjopen-2025-111467 online supplemental file 6

10.1136/bmjopen-2025-111467 online supplemental file 7

## Data Availability

As per the ethics approval, the data from this study will be securely archived for a minimum of 10 years. Any sharing of the data will be subject to review by University of Bristol data access committee. Any queries on how to access the data set should be directed to the corresponding author.
