# Supplementary material for: Malignant upper urinary tract obstruction resulting in hospital admission: a qualitative study of patient, carer and clinician experiences and information received
Source: BMJ Open. 2026 Mar 30;16(3):e111467. doi: 10.1136/bmjopen-2025-111467 (PMC13052715; doi:10.1136/bmjopen-2025-111467)
Supplement: online supplemental file 1 [file bmjopen-16-3-s001.docx]

| 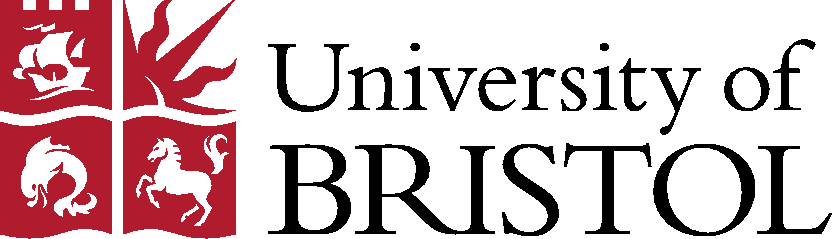 | 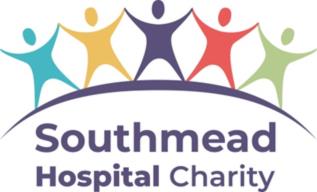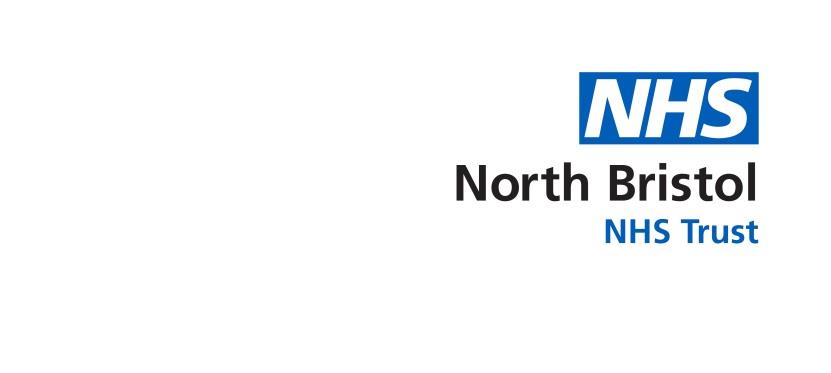 |
| --- | --- |

***Radiology***

A qualitative evaluation of patient, carer and clinician perspectives on Percutaneous Nephrostomy and Ureteric Stenting for Malignant Upper Tract Obstruction (MUTO)

**Topic guide for clinician interviews in hospital or online**

**Introduction:**

Restate that we can pause or stop for any clinical duties, or any other reason. Please say.

Remind that it will be recorded and confidential/anonymised.

Any questions about the study or the interview before we begin?

- I understand that there are two groups of patients, those where the MUTO is picked up in advance and those where they are admitted as an emergency with MUTO. How would you characterise these patients? Could you please describe any differences in approach and clinical decision making between these two groups?
- Do you feel all requests have been appropriately discussed (at a senior enough level, with you, or with clear multidisciplinary team involvement) before referral for urology / IR intervention?
- What’s your impression of the extent to which are patients and their family involved in the decision making?
- How do you feel about your role in the treatment of MUTO? Do you feel included in the multidisciplinary team discussion prior to meeting these patients? Do you ever question whether you are doing the right intervention?
- Have you ever had to have a further discussion about appropriateness of the intervention after reviewing the patient yourself?
- How technically demanding do you find the procedure to do? How commonly would you not be successful?
- What approach do you take to anaesthesia for these patients?
- What are the complications / risks of the procedure as you see them?
- What information do you discuss with patients (is it too late to go into depth when they are in the IR suite)?
- What is your view on the burden of either long term nephrostomies / antegrade stents?
- Do you receive updates on how the patient is tolerating the stent/drain once they have left your care?
- Would it be helpful to have guidance on whether the procedure is appropriate in any particular case? If so in what form and from whom?
- Do you have any other thoughts to share on the issue of nephrostomy and stenting for patients close to the end of their lives?

***Thank you***
