# Supplementary material for: Malignant upper urinary tract obstruction resulting in hospital admission: a qualitative study of patient, carer and clinician experiences and information received
Source: BMJ Open. 2026 Mar 30;16(3):e111467. doi: 10.1136/bmjopen-2025-111467 (PMC13052715; doi:10.1136/bmjopen-2025-111467)
Supplement: online supplemental file 2 [file bmjopen-16-3-s002.docx]

| 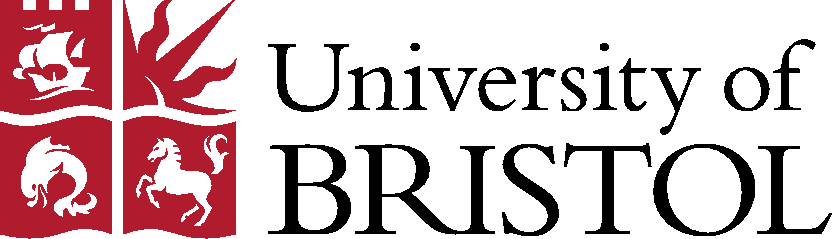 | 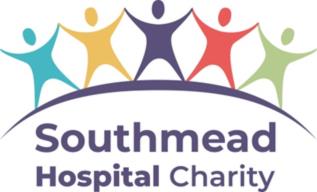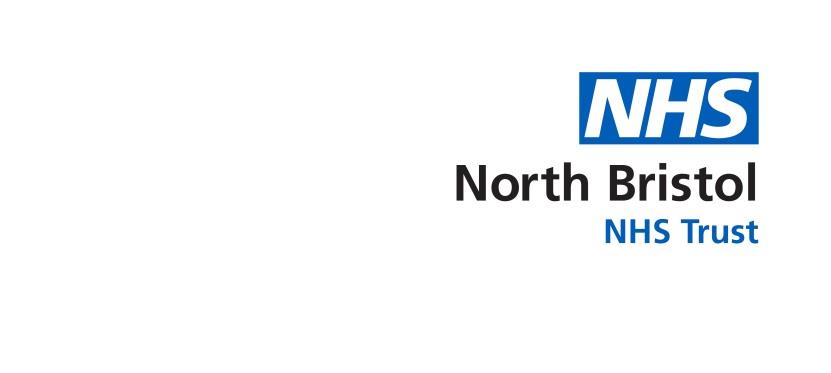 |
| --- | --- |

***Oncologist***

A qualitative evaluation of patient, carer and clinician perspectives on Percutaneous Nephrostomy and Ureteric Stenting for Malignant Upper Tract Obstruction (MUTO).

**Topic guide for clinician interviews in hospital or online**

**Introduction:**

Restate that we can pause or stop for any clinical duties, or any other reason. Please say.

Remind that it will be recorded and confidential/anonymised.

Any questions about the study or the interview before we begin?

- We’re aware in the follow up of cancer patients with obstruction of their urinary tract - How often do you see these patients, what are your concerns when you see it and when would you consider referring your patients to have this treated.
- I understand that there are two groups of patients, those where the MUTO is picked up in advance and those where they are admitted as an emergency with MUTO. Could you please describe any differences in approach and clinical decision making between these two groups?
- Are there any patients for whom you feel these procedures are not appropriate.
- What do you think the benefits are of these treatments for the patient.
- Do you feel all requests have been appropriately discussed (at a senior enough level, with you, or with clear multidisciplinary team involvement) before referral for urology / IR intervention? Does it happen often that your patients are admitted unplanned and how do you feel about decision making in that scenario.
- Are Palliative care colleagues involved in the decision-making process? If so, how?
- To what extent are patients and their family involved in the decision making?
- Are there ever disagreements? What is the nature of these?
- Oncology is clearly a broad speciality when on call do you feel able to offer advice about prognosis to those admitted as an emergency or do you think it should be offered by a specific MDT or by the oncologist who knows the patient best? Why?
- Do you ever discount these procedures on the basis of frailty and/or quality of life considerations? How do you explain this to patients and relatives?
- To what extent do you think that this group of patients need to be managed as an emergency? What are the implications of this?(could they be planned most of the time)
- How do you decide about whether further treatment lines are necessary?
- What are your views on the burden of this intervention (nephrostomy/stent )
- What are the criteria that you have for commencing further treatment after intervention with nephrostomy or stent?
- As a cancer specialist that often manages these patients long term what is your view on the benefits of this procedure?
- Would guidelines be helpful in the management of these patients? If so from whom?
- Do you have any other thoughts to share on the issue of nephrostomy and stenting for patients close to the end of their lives?

***Thank you***
