# Supplementary material for: Malignant upper urinary tract obstruction resulting in hospital admission: a qualitative study of patient, carer and clinician experiences and information received
Source: BMJ Open. 2026 Mar 30;16(3):e111467. doi: 10.1136/bmjopen-2025-111467 (PMC13052715; doi:10.1136/bmjopen-2025-111467)
Supplement: online supplemental file 3 [file bmjopen-16-3-s003.docx]

| 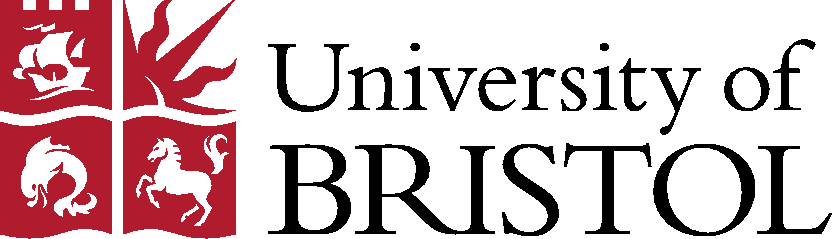 | 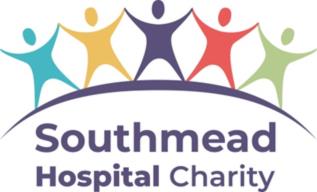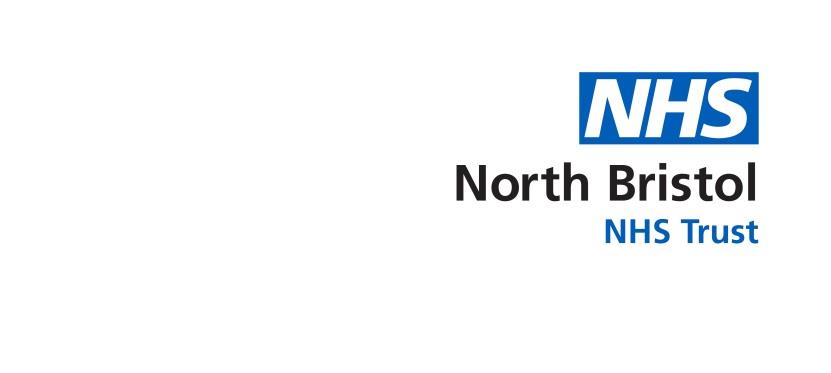 |
| --- | --- |

***Urologist***

A qualitative evaluation of patient, carer and clinician perspectives on Percutaneous Nephrostomy and Ureteric Stenting for Malignant Upper Tract Obstruction (MUTO)

**Topic guide for clinician interviews in hospital or online**

*Introduction:*

Restate that we can pause or stop for any clinical duties, or any other reason. Please say.

Remind that it will be recorded and confidential/anonymised.

Any questions about the study or the interview before we begin?

- What is your approach to patient counselling and treatment decision making in MUTO presenting as an emergency?
- How does the compare to the provision of information and support for decision making for elective referrals?
- When do you consider nephrostomy versus retrograde stent versus no treatment?
- I understand that nephrostomy is performed using local anaesthetic and retrograde stenting under general. As Retrograde Stenting requires GA are there any groups of patients that would be unsuitable to stent. Would they then be offered a nephrostomy and how painful is that? What are your approach to anaesthesia for these procedures?
- Do you ever assess patients for their frailty and what’s your view on the appropriateness of these procedures in frail patients?
- How well do you feel supported by a multidisciplinary team when making decisions, do you seek specialist oncology input? How does this work?
- To what degree are ethical considerations discussed?
- How technically demanding do you find the stent procedure to do? How commonly would you not be successful?
- What are the complications / risks of the procedure as you see them?
- Are you made aware of how well, or otherwise, the patient is as a result of the procedure once they leave your care?
- What is your view on the burden of either long term nephrostomies /stents? Do any other considerations other than clinical appropriateness come into play?
- Would it be helpful to have guidelines for these procedures? If so from whom?
- Do you have any other thoughts on these procedures for very frail patients close to end of life?

***Thank you***
