# Supplementary material for: Malignant upper urinary tract obstruction resulting in hospital admission: a qualitative study of patient, carer and clinician experiences and information received
Source: BMJ Open. 2026 Mar 30;16(3):e111467. doi: 10.1136/bmjopen-2025-111467 (PMC13052715; doi:10.1136/bmjopen-2025-111467)
Supplement: online supplemental file 4 [file bmjopen-16-3-s004.docx]

| 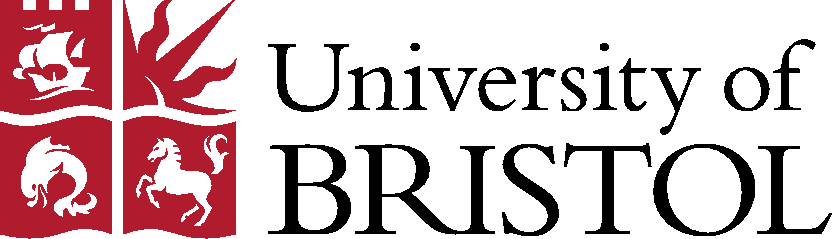 | 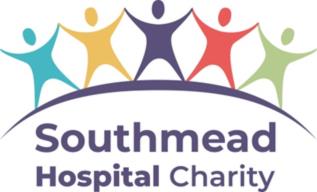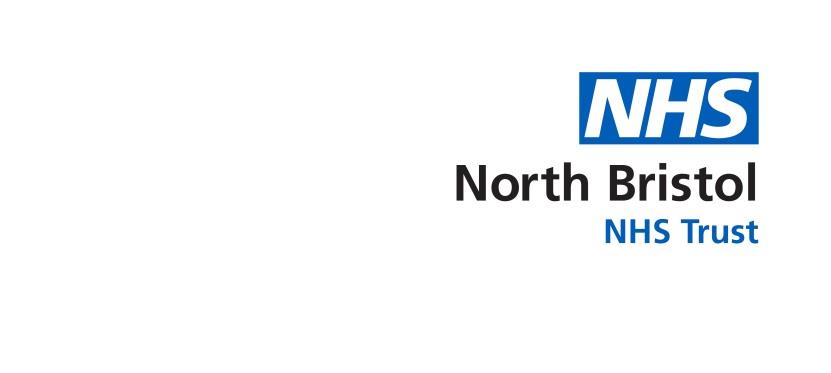 |
| --- | --- |

A qualitative evaluation of patient, carer and clinician perspectives on Percutaneous Nephrostomy and Ureteric Stenting for Malignant Upper Tract Obstruction (MUTO)

**Topic guide for patient interviews at home**

***Safety protocol:*** *Text message Dr Jon Banks before entering the home to let him know that I am entering the patients home and what time I expect to leave.*

**Introduction:**

Good to see them again and thank you for the invitation.

Restate that we can stop completely or stop to rest at any time, please say.

Remind that it will be recorded and confidential/anonymised.

Any questions about the study or the interview before we begin?

**Background:**

- How are you today?
- How have you been getting on since the operation?
- How are you managing with the drain at home?
  - Have you needed any trips back to hospital?
  - Do you have any more treatments planned?
  - What kind of support have you had from the hospital, GP or others?
  - Was this enough?
- How does this affect what you can do? What would you like to be able to do?
- What kind of support do you have at home/away from the hospital?

**Nephrostomy/Stent:**

- Is there any information, either written or from the doctors/nurses, that you would like to have known before the operation
- Did you feel as though you understood what was happening or was anything unclear at the time or since?
- How much was the operation and what happened afterwards what you expected?
- (post op) how are you feeling about it now? Any worries or concerns, unanswered questions?
- Are you pleased that you had the operation to drain your kidney’s, or would you make a different decision with hindsight?
- Is there anything else you’d like to tell me about how this operation and the after effects have been for you?

***Thank you!***

***Safety protocol:*** *On leaving text message Dr Jon Banks to advise him that I am safe.*
