# Supplementary material for: Malignant upper urinary tract obstruction resulting in hospital admission: a qualitative study of patient, carer and clinician experiences and information received
Source: BMJ Open. 2026 Mar 30;16(3):e111467. doi: 10.1136/bmjopen-2025-111467 (PMC13052715; doi:10.1136/bmjopen-2025-111467)
Supplement: online supplemental file 5 [file bmjopen-16-3-s005.docx]

| 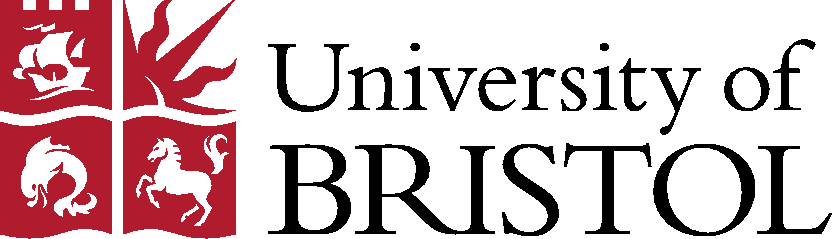 | 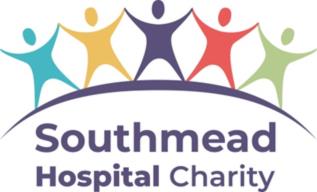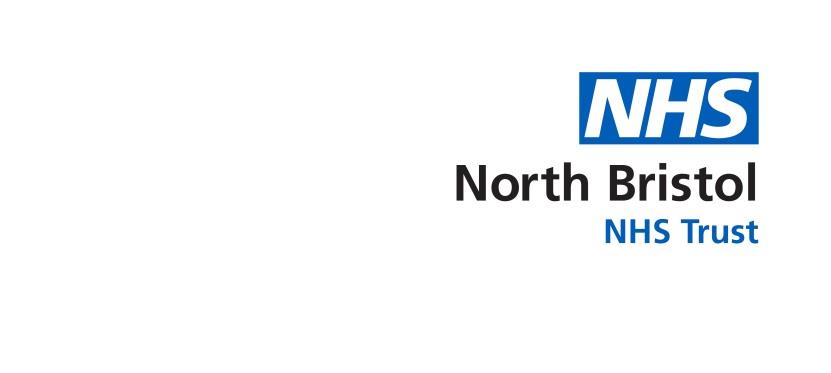 |
| --- | --- |

A qualitative evaluation of patient, carer and clinician perspectives on Percutaneous Nephrostomy and Ureteric Stenting for Malignant Upper Tract Obstruction (MUTO)

**Topic guide for patient interviews in hospital**

**Introduction:**

Restate that we can stop completely or stop to rest at any time, please say.

Remind that it will be recorded and confidential/anonymised.

Any questions about the study or the interview before we begin?

**Background:**

- Please could you tell me a little about your illness,
- what you understand about it and how you’re feeling about things at the moment?
- What treatments have you had so far? Do you have other treatment planned other than the kidney drainage?
- How long have you been in hospital this time?
- How does this affect what you can do? What would you like to be able to do?
- What kind of support do you have at home/away from the hospital?

**Nephrostomy/Stent:** (before or after the procedure)

- I think you’ve had/are going to have an operation to unblock your kidneys, is that correct?
- Can you tell me about how the decision to do that was made? (who’s involved, who they spoke with, time for reflection, asking questions, information)
- Why did you choose to have the operation rather than not? (or other way around if declined)
- Did you feel as though you understood what was happening or was anything unclear?
- (if pre op) is there anything else you would like to find out about before it happens?
- Was/is there anything else you would have liked to know about the operation before it happened?
- Can you describe how the operation was for you?
- Was it what you expected?
- (post op) how are you feeling about it now? Any worries or concerns, unanswered questions?
- Is there anything else you’d like to tell me about how this operation has been for you?

**Thank you for spending the time today talking about this with me.**

I’m hoping to come to visit you at home in a couple of weeks if you feel well enough. Is that’s ok? (Preliminary arrangements if possible)

Is there anything I can do for you now? Nurse? Carer? Water……
