# Supplementary material for: Malignant upper urinary tract obstruction resulting in hospital admission: a qualitative study of patient, carer and clinician experiences and information received
Source: BMJ Open. 2026 Mar 30;16(3):e111467. doi: 10.1136/bmjopen-2025-111467 (PMC13052715; doi:10.1136/bmjopen-2025-111467)
Supplement: online supplemental file 6 [file bmjopen-16-3-s006.docx]

| 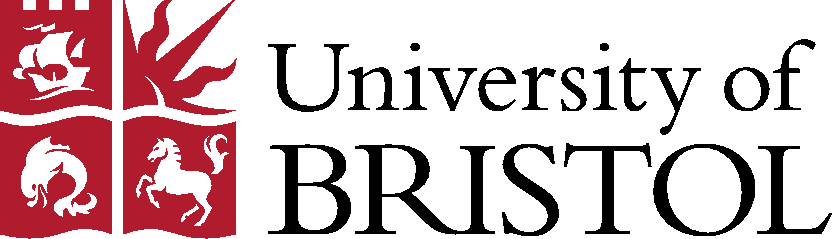 | 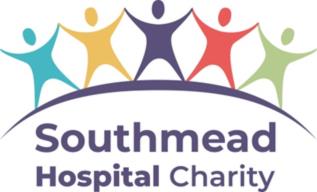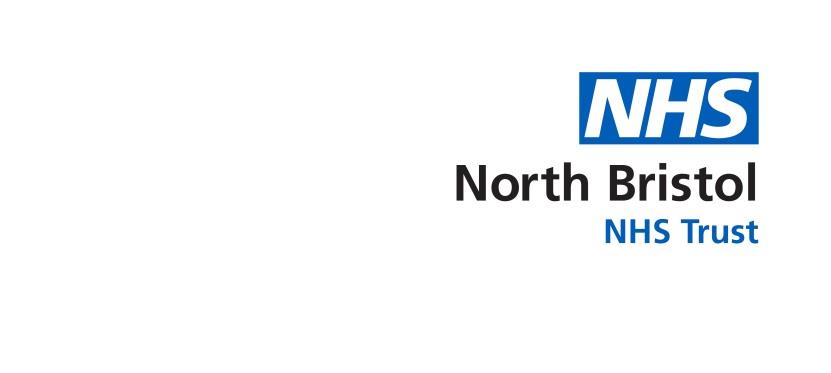 |
| --- | --- |

A qualitative evaluation of patient, carer and clinician perspectives on Percutaneous Nephrostomy and Ureteric Stenting for Malignant Upper Tract Obstruction.
_______________________________________________________________

**Topic guide for carer interviews in hospital or at home**

**Safety protocol:** Text message Dr Jon Banks before entering the home to let him know that I am entering the patients home and what time I expect to leave.

**Introduction:**

Restate that we can stop completely or stop to rest at any time, please say.

Remind that it will be recorded and confidential/anonymised.

Any questions about the study or the interview before we begin?

1. How has caring for (name) been for you?
2. Can you remember how the suggestion to have the kidney drainage came about?
   1. Did you and (name) talk about it? What did you think about it?
   2. Any concerns? Any positives? Any pressure?
   3. Were you given any written information? How helpful was it?
   4. Did you have any opportunities to ask questions if you had any?
   5. Which doctors or nurses did you talk to about this?
3. Who made the decision to have/not have the operation? How was it made?
4. How was the operation for (name)?
5. How has recovering from the operation been?
6. Is there anything, with hindsight, that you would have liked to have been told about/had information about before the operation? Why?
7. How has dealing with the drain (Jon, other physical issues here) been since?
8. Are there any follow up visits planned or other further intervention?
9. Would you make the same decision again?
10. What do you understand (name’s) treatment to be from now on?
11. Is there anything else you’d like to add that we haven’t covered?

***Thank you!!***

**Safety protocol:** On leaving text message Dr Jon Banks to advise him that I am safe.
