# Supplementary material for: Malignant upper urinary tract obstruction resulting in hospital admission: a qualitative study of patient, carer and clinician experiences and information received
Source: BMJ Open. 2026 Mar 30;16(3):e111467. doi: 10.1136/bmjopen-2025-111467 (PMC13052715; doi:10.1136/bmjopen-2025-111467)
Supplement: online supplemental file 7 [file bmjopen-16-3-s007.docx]

| 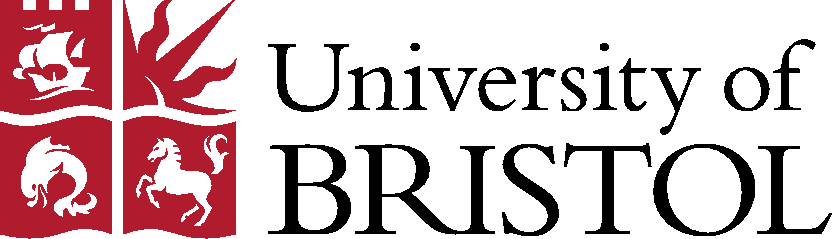 | 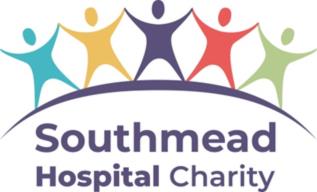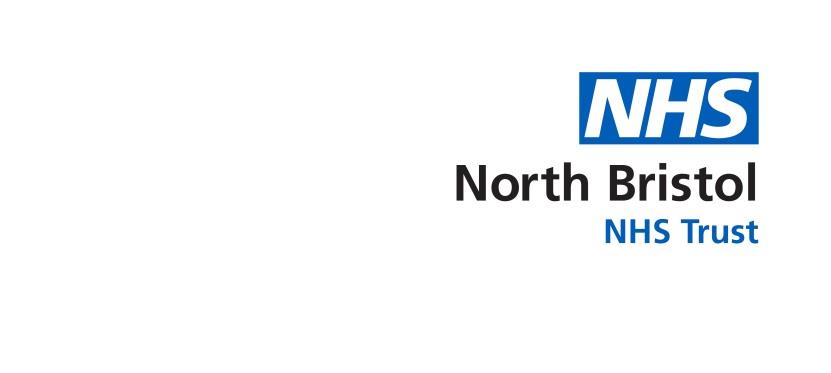 |
| --- | --- |

***Nursing Team***

**A qualitative evaluation of patient, carer and clinician perspectives on Percutaneous Nephrostomy and Ureteric Stenting for Malignant Upper Tract Obstruction (MUTO)**

**_________________________________________________________________________________________________________________________________________________________________________________________________________________________________**

***Topic guide for clinician interviews in hospital or online***

**Introduction:**

Restate that we can pause or stop for any clinical duties, or any other reason. Please say.

Remind that it will be recorded and confidential/anonymised.

Any questions about the study or the interview before we begin?

Please could you describe your role in the department? How long have you been doing this?

Considering frail patients who may be coming towards the end of life:

- What are your observations on how management decisions on these types of patients are made?
- What levels of support do you feel able to offer?
- Is there anything that you think patients and/or relatives struggle with before or after the procedure?
- Have you ever felt that it was inappropriate to be doing the procedure? If so what were your concerns? Were you able to raise these with anyone?
- What other avenues of support do these patients have? How effective are these do you think?
- What do you think the impact of intervention carries for these patient groups?
- How often are you faced with discharge issues because of the intervention?
- Is there anything else about this procedure in frail and end of life patients that you would like to add?

***Thank you***
